# Reply to LT Cacau and DM Marchioni

**DOI:** 10.1093/ajcn/nqac007

**Published:** 2022-04-01

**Authors:** Anna Stubbendorff, Emily Sonestedt, Stina Ramne, Isabel Drake, Elinor Hallström, Ulrika Ericson

**Affiliations:** From Department of Clinical Sciences Malmö, Lund University, Malmö, Sweden; From Department of Clinical Sciences Malmö, Lund University, Malmö, Sweden; From Department of Clinical Sciences Malmö, Lund University, Malmö, Sweden; From Department of Clinical Sciences Malmö, Lund University, Malmö, Sweden; Department of Agriculture and Food, Research Institutes of Sweden (RISE), Lund, Sweden; From Department of Clinical Sciences Malmö, Lund University, Malmö, Sweden

Dear Editor:

We thank Cacau and Marchioni for their interest in our article on the development of an EAT-Lancet index and its relation to mortality (1). Dietary indexes can be constructed in many different ways and, although our index and the Planetary Health Diet index by Cacau et al. (2) both aimed to capture adherence to the EAT-Lancet diet, they differed with regard to the scoring method and interpretation of proposed reference intakes (3). However, we apologize for not highlighting that the index developed by Cacau et al. (2) also is constructed as a score that gradually captures intake variation outside proposed reference levels of the EAT-Lancet diet, in contrast to binary scores (4, 5). Including this information in our discussion would have contributed to a more complete picture of current dietary indexes developed to reflect adherence to the EAT-Lancet diet.

We would also like to take the opportunity to express the need for comparison of the different indexes. We acknowledge that there is a need for more studies with the different indexes that have been developed, including the index by Cacau et al. (2), to evaluate strengths and weaknesses, such as the inclusion of binary food components compared with multi-level components, absolute intake components compared with energy-adjusted components, and whether certain components should be defined as emphasized or limited. We have examined the association between our EAT-Lancet index and mortality, and it would be very interesting to compare different scores when it comes to health outcomes in different populations.

## Notes

This original work was supported by the Swedish Heart-Lung Foundation (nr 20200482), Crafoord Foundation (nr 20210674), and Agenda 2030 Graduate School, Lund University.

No funding agencies had any influence on this reply.
